# Supplementary material for: Shape matters: inferring the motility of confluent cells from static images
Source: Soft Matter. 2025 Jun 24;21(33):6504–15. doi: 10.1039/d5sm00222b (PMC12235246; doi:10.1039/d5sm00222b)
Supplement: SM-021-D5SM00222B-s001 [file SM-021-D5SM00222B-s001.pdf]

Supplementary Information (SI) for Soft Matter.
This journal is © The Royal Society of Chemistry 2025

Supplementary material for "Shape matters: Inferring the motility of confluent cells from static images"

Quirine J. S. Braat,[1,*] Giulia Janzen,[1,2,*] Bas C. Jansen,[1,*] Vincent E. Debets,[1] Simone Ciarella,[3,4] and Liesbeth M. C. Janssen[1,5,†]

[1]*Department of Applied Physics, Eindhoven University of Technology, P.O. Box 513, 5600 MB Eindhoven, The Netherlands*
[2]*Department of Theoretical Physics, Complutense University of Madrid, 28040 Madrid, Spain*
[3]*Netherlands eScience Center, Amsterdam 1098 XG, The Netherlands*
[4]*Laboratoire de Physique de l'Ecole Normale Supérieure, ENS, Université PSL, CNRS, Sorbonne Université, Université de Paris, F-75005 Paris, France*
[5]*Institute for Complex Molecular Systems, Eindhoven University of Technology, P.O. Box 513, 5600 MB Eindhoven, The Netherlands*

SIMULATION PARAMETERS

Symbol	Value	Explanation
(x, y)	(300, 300)	Domain size of the box (periodic boundary conditions)
N	144	Total number of cells
N_a	varied	Total number of active cells
T_m	10	Temperature
$\mathrm{O}_{\text{pixel-copy}}$	2	Neighbour order for the pixel-copy attempts
O_{adh}	8	Neighbour order for the adhesion term
$\mathrm{O}_{\text{boundary}}$	1	Neighbor order for the boundary pixel counting
V_t	625	Target volume of the cells
λ_{vol}	1	Strength of the volume constraint
S_t	150	Target surface of the cells
λ_{sur}	1 (or 0)	Strength of the surface constraint (depends on the perimeter of a cell)
κ_i	varied	Magnitude of the motility of the cells. The index i indicates whether the cells is active or passive
J_{AA}	5	Adhesion for two neighboring active cells
J_{AP}	5	Adhesion for a neighboring passive and active cell
J_{PP}	5	Adhesion for two neighboring passive cells

TABLE S1: Overview of the simulation parameters used in the Cellular Potts Model.

VISUALIZATION OF THE FEATURES

The features that are extracted from the simulations are described in Table 1 in the manuscript. Figure S1 shows a visualization of the variables defined in the table. Panel A shows the definition of the centre of mass and the pixel vector $\vec{r}_i$ that describes the position of the pixel i with respect to the centre of mass. These variables are used to calculate the volume and moments of mass of the cells. Panel B shows the definition of the ellipse that is fitted to the cells. The ellipse can be described by the semi-major axis a, semi-minor axis b and an orientation θ. Panel C shows the definition of the variables necessary to calculate the bond-order parameters. R_j is the centre-of-mass connection between a central cell and its neighbour j. Moreover, it shows the definition of the bond length β_j. The bond length

* These authors contributed equally to this work.
† l.m.c.janssen@tue.nl

is determined using the common surface method as implemented in CompuCell3D [2]. Panel D shows the definition of the alignment between neighbouring cells and combines the ellipse characteristics (see panel B) of the central cell and its neighbour j. Finally, panel E shows the boundary pixels (in red) extracted using the build-in boundary pixel detection in CompuCell3D [2].

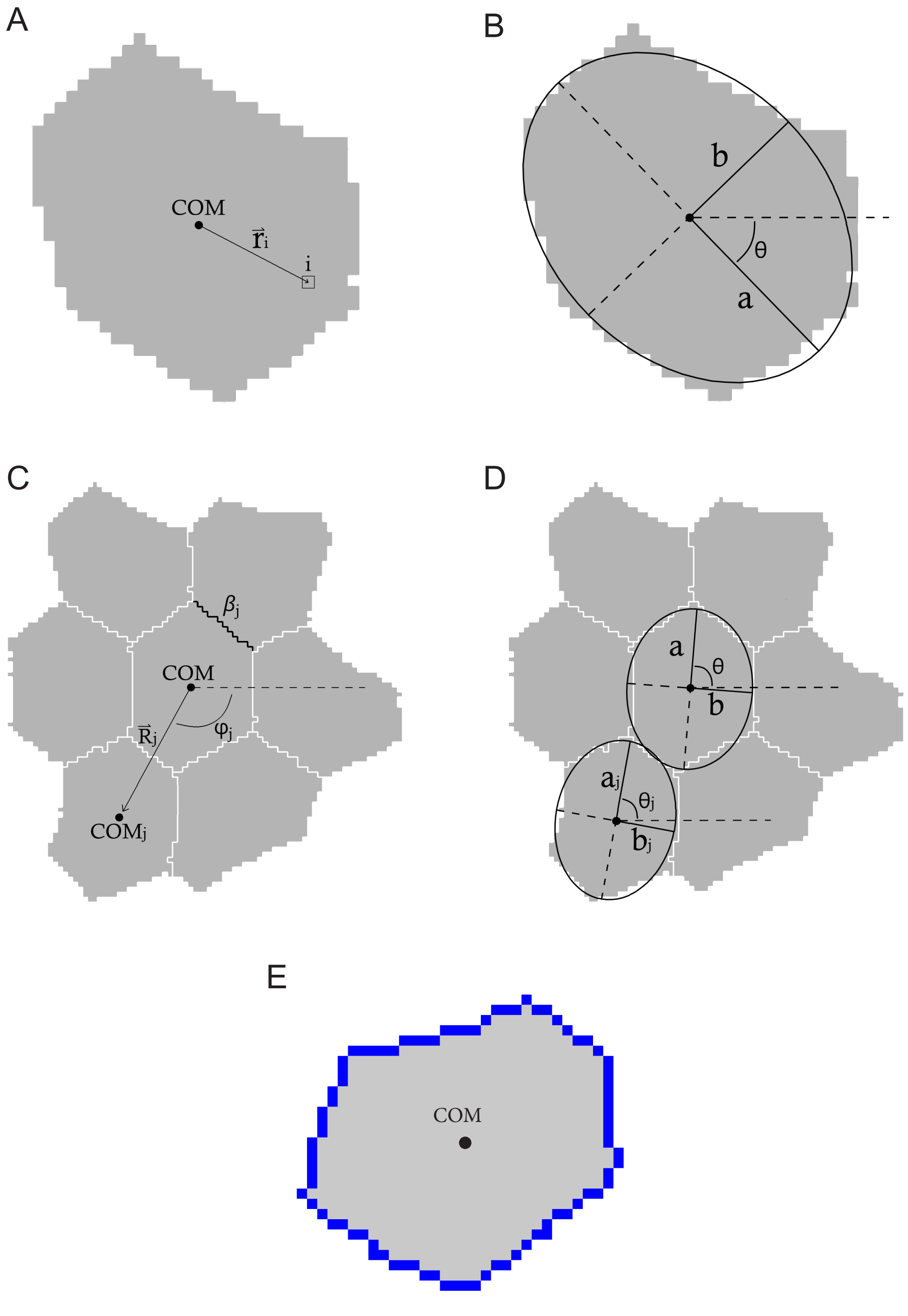

FIG. S1: Visualization of the definition of the variables used in the definition of the features. Panels A-E show the different properties extracted from the cell and its neighbours. The properties are extracted using the built-in functionality in CompuCell3D [2].

SHAPE CHARACTERISATION

During the feature calculation, cell properties are extracted for all cells in the simulation. We fit an ellipse to all cells to get a first estimate for the cell's shape. Based on the semi-major axis and the semi-minor axis of the ellipse, we can calculate the aspect ratio as the ratio between the two. Fig. S2 shows the aspect ratio for the non-motile cells ($\kappa_p = 0$) and the motile cells ($\kappa_a = 1500$) as we vary the number of motile cells in the confluent layer. For a single motile cell, the aspect ratio of the motile cells is significantly larger than for the non-motile cells. This difference becomes smaller as the number of motile cells increases. For $N_a = 60$, we observe that the mean aspect ratio for the non-motile cells is even larger than for the motile ones. While the mean aspect ratio of the motile cells hardly changes, the shape of the non-motile cells is more strongly affected by the presence of the motile cells. We observe similar trends for the bond order parameter ψ_6, which measures the presence of a six-fold symmetry around cells (see Fig. S4).

When both cell types are motility, but the motility is different (high-motility cells have $\kappa_a = 300$ and the low-motility cells have $\kappa_p = 150$), the differences between the two cell types are less apparent (see Fig. S3 and S5). It becomes more difficult to distinguish the two cell types. The machine-learning results support these observations.

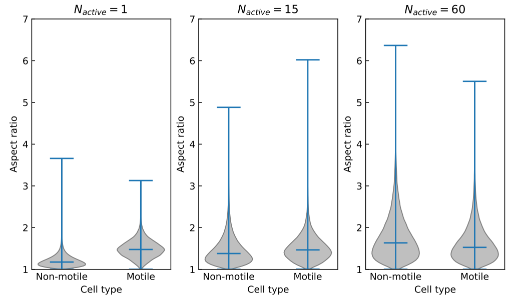

FIG. S2: Aspect ratio for the active and passive cells in the simulation for $\kappa_p = 0$ and $\kappa_a = 1500$. The results are shown (left to right) for 1, 15 and 60 active cells in the simulations. As the number of active cells increases, the aspect ratios become more similar.

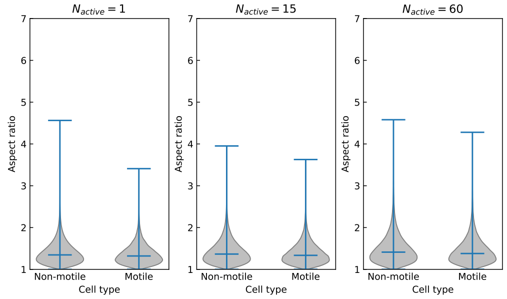

FIG. S3: Aspect ratio for the active and passive cells in the simulation for $\kappa_p = 150$ and $\kappa_a = 300$. The results are shown (left to right) for 1, 15 and 60 active cells in the simulations. The aspect ratios for both cell types are very similar.

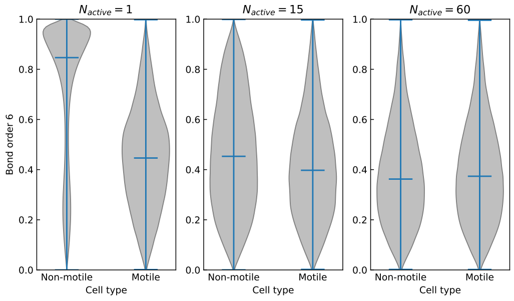

FIG. S4: Bond order parameter ψ_6 for the active and passive cells in the simulation for $\kappa_p = 0$ and $\kappa_a = 1500$. The results are shown (left to right) for 1, 15 and 60 active cells in the simulations. For $N_a = 1$, the passive cells are organized in a six-fold symmetry. As the number of active cells increases, the underlying structure around active and passive cells becomes similar.

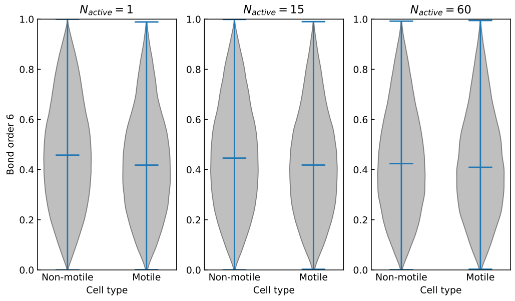

FIG. S5: Bond order parameter ψ_6 for the active and passive cells in the simulation for $\kappa_p = 150$ and $\kappa_a = 300$. The results are shown (left to right) for 1, 15 and 60 active cells in the simulations. The cells does not show a strong six-fold symmetry. When the motility of the high-motility and low-motitily cells becomes more similar, so does the bond order parameter ψ_6.

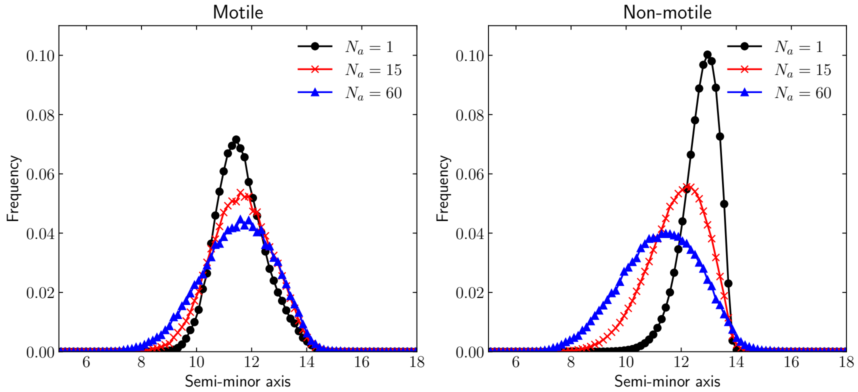

FIG. S6: Distribution of the semi-minor axis for the motile (left) and non-motile (right) cells as the number of motile cells in the confluent layer changes. The results are normalized for a fair comparison between the number of motile and non-motile cells. We have set $\kappa_a = 1500$ and $\kappa_p = 0$.

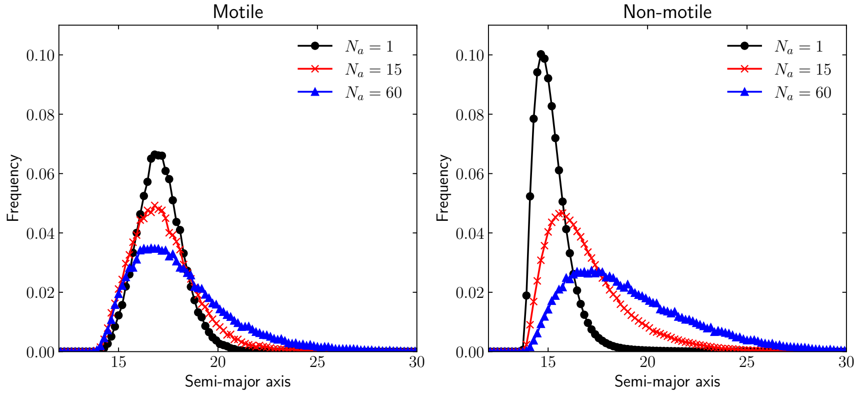

FIG. S7: Distribution of the semi-major axis for the motile (left) and non-motile (right) cells as the number of motile cells in the confluent layer changes. The results are normalized for a fair comparison between the number of motile and non-motile cells. We have set $\kappa_a = 1500$ and $\kappa_p = 0$.

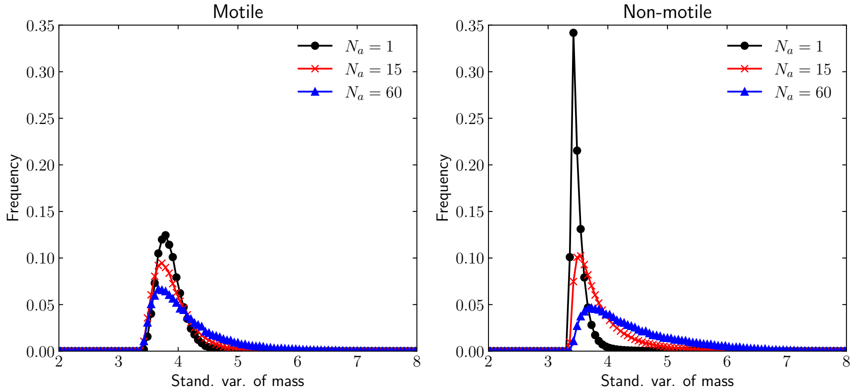

FIG. S8: Distribution of the standard variance of mass for the motile (left) and non-motile (right) cells as the number of motile cells in the confluent layer changes. The results are normalized for a fair comparison between the number of motile and non-motile cells. We have set $\kappa_a = 1500$ and $\kappa_p = 0$.

Second, we measured the distribution of the semi-major axis and the semi-minor axis in a different way. As the number of motile cells changes, we may expect that the shape characteristics of the cells change. Figure S6 shows how the distribution of shape characteristics (semi-minor axis here, but similar results for e.g. the semi-major axis in Figure S7) change as N_a changes. The accuracy drops as N_a increases, which could be explained by the fact that the shape characteristics are more similar. There are two interesting observations to highlight: 1) the distribution of the motile cells has a similar shape, but the distribution becomes wider as N_a increases, and 2) the distribution of the non-motile cells changes significantly skewed distribution towards a more symmetric distribution similar to the distribution of the motile cells as N_a increases. These graphs can explain why the accuracy drops as N_a increases. These results may also explain why the generalization is poor when $N_a = 1$ or $N_a = 60$ as these distributions are very different, but works better when the number of motile cells is in between these values.

Third, we have also plotted the standard variation in mass in Figure S8. Here, the distributions for the motile and non-motile cells remain different, even for larger N_a and therefore this could be a characteristic feature that has an important role in the predictive power of the ML model as the cells become more similar. This features also appears in the SHAP and PCA analysis.

Finally, Figure S9 shows additional structural features for varying N_a, γ. These results support the observations that the ML predictions become more difficult for increasing N_a and γ. Similarly, some of the non-local shape features (see Figure S10) are nearly identical, indicating that averaging properties over the local neighbourhood leads to a significant reduction of the available information. Others, on the other hand, show more differences and may have a stronger potential for predictive power. The local shape features appear to show the largest differences in the feature distribution for increasing for N_a and γ. Nonetheless, even within the subset of local shape features, some features have more distinct differences than others. These results indicate that it may be difficult to determine whether the cells belong to the high-motility or low-motility phenotype based on individual distributions, and that the ML model can learn the more complex dependencies between these features to make accurate predictions of cells on the individual level.

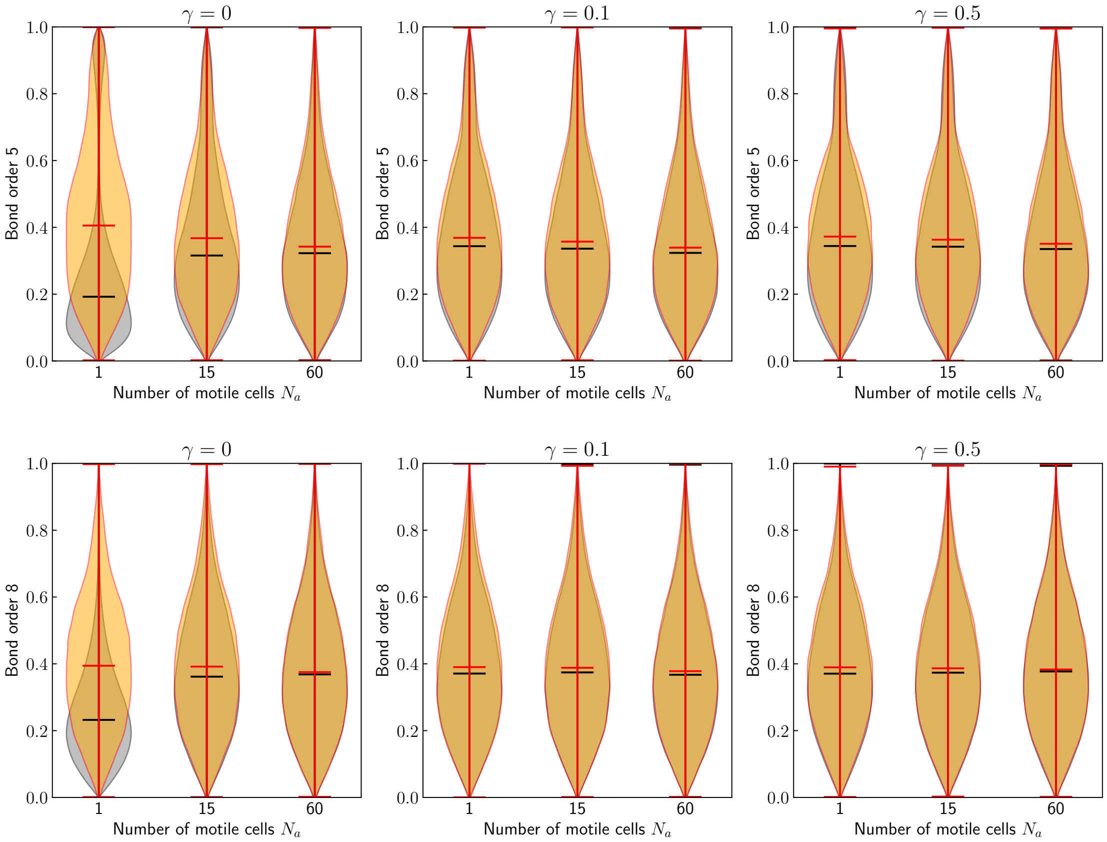

FIG. S9: Subset of the distributions of structural features extracted from the simulations. Bond order parameter ψ_5 (top-row) describes the five-fold symmetry around cells, and the bond order parameter ψ_8 (bottom-row) describes the eight-fold symmetry. The coloured (red) plots are for the motile cells, and the gray plots are for the zero-motility (left) and low-motility (middle, right). The distributions are very similar, except for $\gamma = 0$, $N_a = 1$.

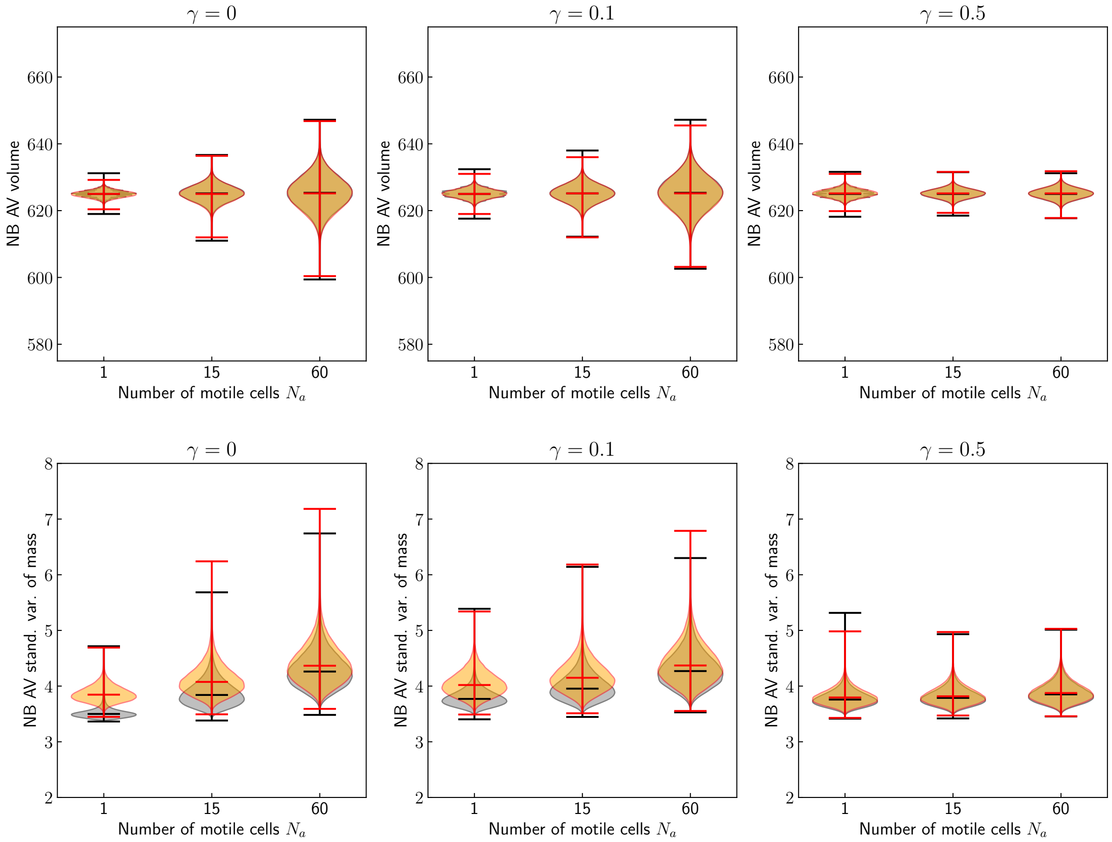

FIG. S10: Subset of the distributions of non-local (neighbour) shape features extracted from the simulations. The volume of the cells and the X are shown in the top-row and bottom-row, respectively. The coloured (red) plots are for the motile cells, and the gray plots are for the zero-motility (left) and low-motility (middle, right). The distributions differ in their potentially predictive power.

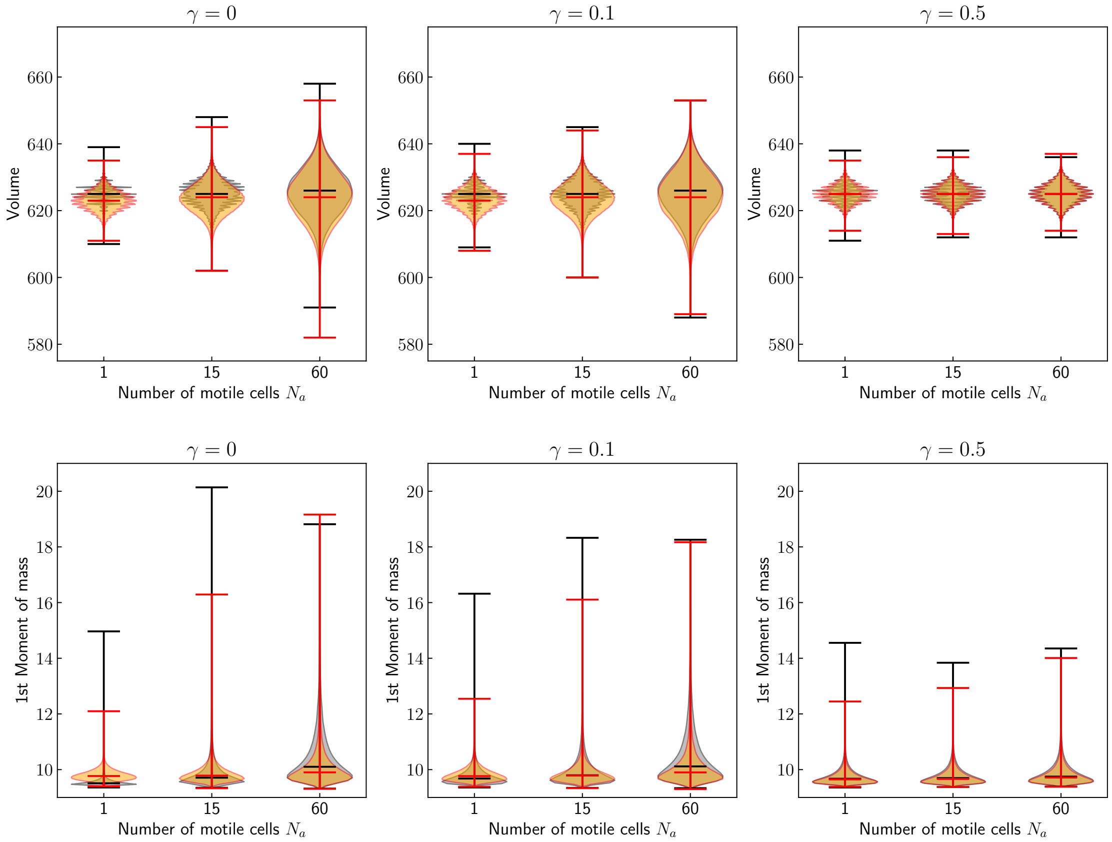

FIG. S11: Subset of the distributions of local shape features extracted from the simulations. The volume of the cells and the X are shown in the top-row and bottom-row, respectively. The coloured (red) plots are for the motile cells, and the gray plots are for the zero-motility (left) and low-motility (middle, right). The distributions are different over a larger range of γ and N_a.

SHAPE AND STRUCTURE FEATURES

In Sec. III A, we show the accuracy of the ML prediction when the ML algorithm only uses shape features as input. Fig. S12 shows the accuracy for a ML model that is trained on either the structure features or the shape features. The structure features mainly consider bond order parameters and the distances between neighbouring cells and leads to a significantly worse phenotype prediction. Although the set of structure features is by no means complete, structure features are more difficult to extract from experimental results, as the cell's centre-of-mass can be defined in various ways. Shape features, on the other hand, can be measured directly from static images using image processing.

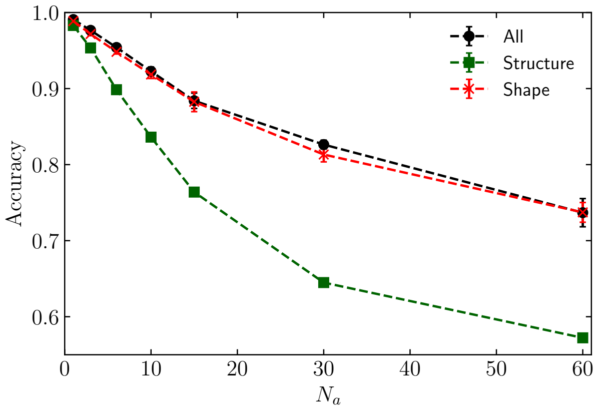

FIG. S12: Accuracy of the ML prediction as the number of active cells N_a changes. The passive cells are non-motile ($\kappa_p = 0$) and the active cells are motile ($\kappa_a = 1500$). The algorithm is trained on the full set of features (black dots), and on a subset of the features, namely 1) structure features (green squares) or 2) shape features (red stars).

COMPARISON OF INCORRECT PREDICTIONS

Apart from the accuracy, we can also study the number of incorrect predictions. These are either false positives (incorrectly identifying a motile cell as non-motile) or false negatives (incorrectly identifying a motile cell as non-motile). Figure S13 shows the number of incorrect predictions of each type for the machine-learning models trained on either all features or solely local shape features. When all features are used, the model predicts approximately the same number of false positives and false negatives. However, since we use only local shape features, the overall accuracy remains the same but for large values of N_a, the model identifies motile cells more often inaccurately while it performs better at predicting the non-motile cell.

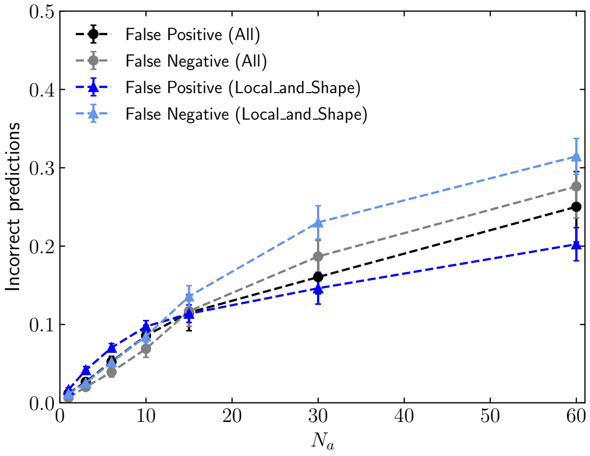

FIG. S13: The number of incorrect prediction for the two machine learning models (in black with all features, in blue with local shape features) corresponding to the result of Figure 3 in the manuscript. The False Positives indicate the instances where a non-motile cell is identified as motile, while the False Negative indicate instances where the motile cell is identified as non-motile. Depending on the features, the number of false positives and false negatives changes.

HEAT MAP FULL SET OF FEATURES

In the Cellular Potts simulations, both the motility strength of both cell types and the number of motile cells varied. Figure S14 shows the difference in accuracy between the learning with the local shape features and the complete set of 145 features. We observe the largest difference between the two neural networks for a small number of active cells. In this regime, using all features is beneficial when the motility of the passive and active cells is similar (γ increases). The larger predictive power of the full set of features, when N_a is small, implies that distortions in the structural features are more distinct in this regime. This effect is also observed in Fig. S12 for small ϕ_a; in this regime, the structural features also allow for accurate phenotype predictions.

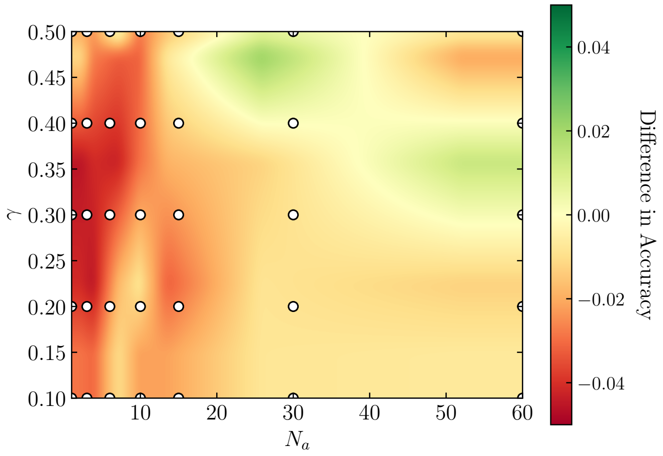

FIG. S14: Heat map of the difference in accuracy between the learning with solely local shape features and the learning with all features. The results are shown for the simulation with a passive force strength ($\kappa_p = 150$). The red indicates the regions in which the prediction with all features is better, and the green indicates the regions in which the predictions with the local shape features are better. Overall, the differences between the two approaches are small.

DYNAMICS

By changing the number of active cells and the active force strength κ_a, the dynamic behaviour of the cells changes. We calculate the effective long-time diffusion coefficient D_{eff} from the mean square displacement. The ratio between the effective diffusion coefficient of active and passive cells, $D_{eff,A}/D_{eff,P}$ correlates with the accuracy, see Fig. S15. When the motility of the active and passive cells are very similar (small ratio $D_{eff,A}/D_{eff,P}$), we observe that the prediction worsens. The predictions are best when the passive cells are non-motile (black).

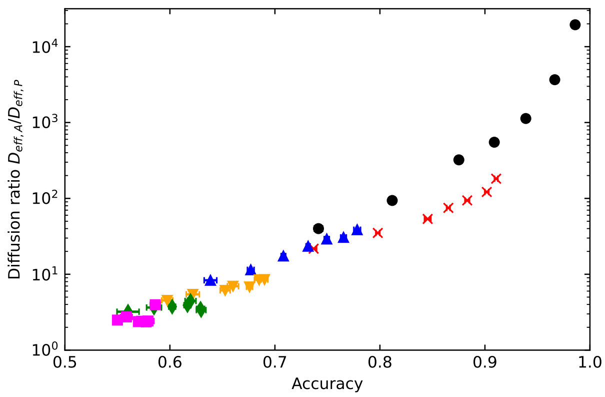

FIG. S15: Correlation between the ratio of diffusion coefficient ($D_{eff,A}/D_{eff,P}$) and the accuracy. Plot for LOCAL AND SHAPE features. The accuracy gets better as the difference in diffusion coefficient becomes larger. The black points ($\kappa_p = 0$) represent a different situation than the coloured points ($\kappa_p = 150$).

ALTERNATIVE MACHINE-LEARNING APPROACHES

Table S2 shows the comparison of the classification accuracies of the model used in the manuscript (a multilayer perceptron, denoted as NN), gradient-boosting, and logistic regression. The performance of the neural network and gradient-boosting models is very similar – they differ only at the second decimal place. In contrast, the logistic regression model consistently yields lower accuracy compared to the other ML methods. The model was implemented in PyTorch [1] and trained using BCEWithLogitsLoss, ensuring numerical stability and proper gradient flow.

We have also applied stratified 5-fold cross-validation with shuffled splits to obtain a more systematic and statistically reliable estimate of the model's performance. This method preserves class balance within each fold and mitigates potential biases arising from data ordering. The accuracy obtained through cross-validation closely matched the average accuracy of the 20 independently trained models, with differences ranging from 0.01 to 0.07.

γ	N_a	NN accuracy	Gradient-boosting accuracy	Logistic regression accuracy	Cross-validation
0.1	1	0.94	0.99	0.73	0.98
0.1	60	0.74	0.77	0.60	0.75
0.5	1	0.60	0.63	0.50	0.56
0.5	60	0.55	0.57	0.5	0.62

TABLE S2: Comparison of the classification accuracies of the model used in the manuscript (a multilayer perceptron, denoted as NN), gradient-boosting, and logistic regression. A cross-validation has been included as well. The results are for four representative parameter sets, but we observe the same trends for other values of N_a and γ.

FEATURE SELECTION: SHAP AND PCA

Figure S16 displays the list of the six most important features selected by SHAP for four different configurations: (a) $N_a = 1$ and $\gamma = 0$ (b) $N_a = 60$ and $\gamma = 0$ (c) $N_a = 1$ and $\gamma = 0.5$ (d) $N_a = 60$ and $\gamma = 0.5$. To assess whether these features are sufficient for accurately classifying cell motility, we train a neural network for each configuration using the six most important features selected by SHAP as input features. For $N_a = 1$ and $\gamma = 0$, the accuracy on the test set using the features shown in Fig. S16 (a) is 0.98, while the accuracy with all features is 0.99. When $N_a = 60$ and $\gamma = 0$, the accuracy with the features from Fig. S16 (b) is 0.66, compared to 0.73 with all features. For $N_a = 1$ and $\gamma = 0.5$, the accuracy with the features in Fig. S16 (c) is 0.60, and the accuracy with all features is 0.607. Lastly, for $N_a = 60$ and $\gamma = 0.5$, the accuracy with the features in Fig. S16 (d) is 0.54, while the accuracy corresponding to all the features is 0.55.

Additionally, PCA analysis was performed with two principal components. Table S3 presents the top ten features contributing the most to the first two principal components for four different configurations: $N_a = 1$ and $\gamma = 0$, $N_a = 60$ and $\gamma = 0$, $N_a = 1$ and $\gamma = 0.5$, and $N_a = 60$ and $\gamma = 0.5$. To evaluate the efficiency of these features for accurately classifying cell motility, we trained a neural network for each configuration using the first two principal components. For $N_a = 1$ and $\gamma = 0$, the variance associated with the first two principal components is 0.54, and the accuracy associated with a neural network trained with these components is 0.96. When $N_a = 60$ and $\gamma = 0$, the variance for the first two principal components is 0.30, and the accuracy for a neural network trained with these components is 0.6. For $N_a = 1$ and $\gamma = 0.5$, the variance related to the first two principal components is 0.30, and the accuracy linked to a neural network trained with these components is 0.52. Lastly, when $N_a = 60$ and $\gamma = 0.5$, the variance associated with the first two principal components is 0.31, and the accuracy related to a neural network trained with these components is 0.53.

These results show that both PCA and SHAP do not outperform the accuracy achieved by a neural network trained with local shape features as input. Furthermore, as explained in the main text, the list of the most important features obtained from these analyses comprises a combination of shape and structural features, making these analyses less computationally efficient.

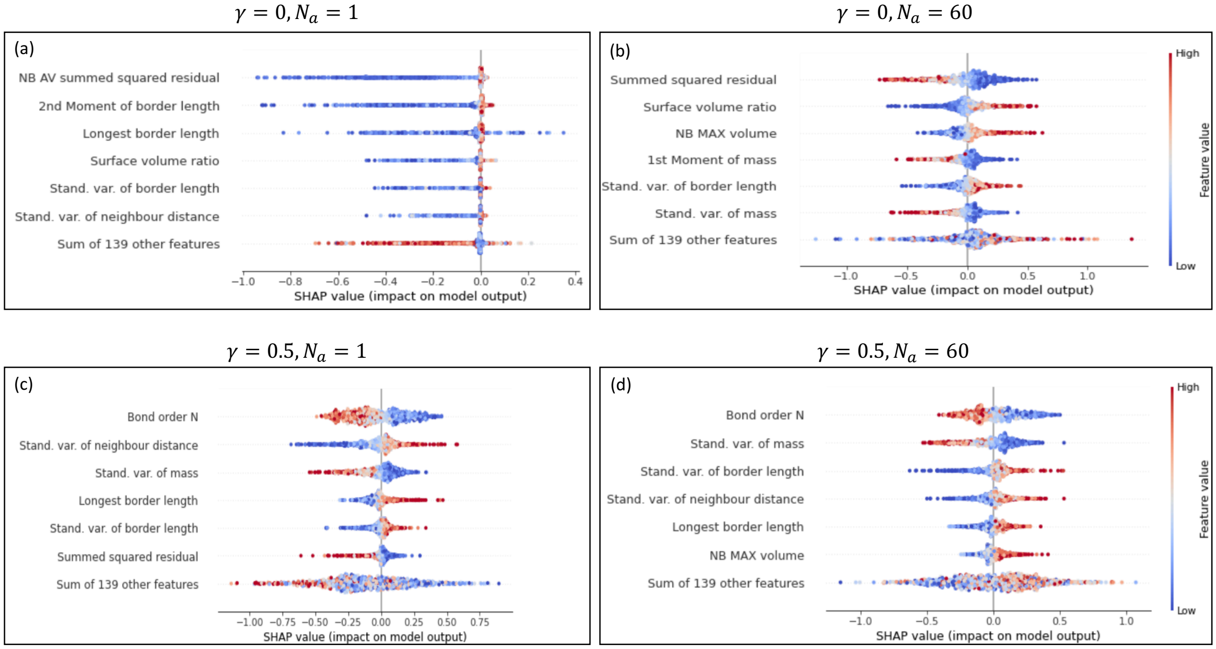

FIG. S16: Interpretation of the multilayer perceptron predictions using SHAP for different combinations of N_a and γ. The SHAP beeswarm plots show the impact of the six most important features on the model's output. The horizontal position (x-axis) of the dots is determined by the SHAP values of the features, while colour is employed to represent the original values of the features. Panels (a), (b), (c), and (d) correspond to different combinations of N_a and γ: (a) $N_a = 1$ and $\gamma = 0$ (b) $N_a = 60$ and $\gamma = 0$ (c) $N_a = 1$ and $\gamma = 0.5$ (d) $N_a = 60$ and $\gamma = 0.5$.

N_a	γ	**Top ten most important features**
1	0	Number of neighbours<br>NB MIN 1st Moment of neighbour distance<br>NB MIN 2nd Moment of neighbour distance<br>1st Moment of neighbour distance<br>1st Moment of border length<br>NB AV 1st Moment of neighbour distance<br>2nd Moment of neighbour distance<br>2nd Moment of border length<br>NB AV 2nd Moment of neighbour distance<br>NB MAX 1st moment of border length
60	0	Standard variation of mass<br>Semi-major axis<br>1st Moment of mass<br>2nd Moment of mass<br>3rd Moment of mass<br>Skewness of mass<br>Surface<br>Surface volume ratio<br>NB AV standard variation of mass<br>Standard variation of neighbour distance
1	0.5	Standard variation of mass<br>1st Moment of mass<br>2nd Moment of mass<br>Skewness of mass<br>3rd Moment of mass<br>Semi-major axis<br>Standard variation of neighbour distance<br>2nd Moment of neighbour distance<br>NB AV 2nd Moment of neighbour distance<br>NB AV standard variation of mass
60	0.5	Standard variation of mass<br>1st Moment of mass<br>2nd Moment of mass<br>Semi-major axis<br>Skewness of mass<br>3rd Moment of mass<br>Standard variation of neighbour distance<br>NB AV standard variation of mass<br>NB AV 1st Moment of mass<br>2nd Moment of neighbour distance

TABLE S3: Top ten features with the greatest contribution to the first 2 principal components extracted by PCA for various combinations of N_a and γ. The formulas used to compute these features are presented in Table 1 of the main text.

REFERENCES

[1] Adam Paszke et al. "Automatic differentiation in PyTorch". In: *NIPS-W* (2017).

[2] Maciej H. Swat et al. *CompuCell3D Manual - version 4.3.1*. 2022.
